# Molecular Characterization of the Myoliquefactive Fish Parasite *Kudoa mirabilis* (Cnidaria, Kudoidae) from SW Indian Ocean and Its Phylogenetic Relationship with the *Kudoa thyrsites* Species Complex

**DOI:** 10.3390/microorganisms8091352

**Published:** 2020-09-04

**Authors:** Lucilla Giulietti, Egil Karlsbakk, Paolo Cipriani, Salome Daniel Shayo, Julia E. Storesund, Arne Levsen

**Affiliations:** 1 Section of Contaminants and Biohazards, Institute of Marine Research (IMR), 5005 Bergen, Norway; Egil.Karlsbakk@uib.no (E.K.); paolo.cipriani@hi.no (P.C.); julia.storesund@hi.no (J.E.S.); arne.levsen@hi.no (A.L.); 2 Department of Biological Sciences (BIO), University of Bergen, 5007 Bergen, Norway; 3 Dar es Salaam Research Center, Tanzania Fisheries Research Institute (TAFIRI), Dar es Salaam 9750, Tanzania; salomeshayo@tafiri.go.tz

**Keywords:** *Kudoa mirabilis*, fish parasite, molecular characterization, myoliquefaction, ‘soft flesh’, allometric characters, *Kudoa thyrsites* complex, sister species, type host

## Abstract

Myxosporean parasites of the genus *Kudoa* are fish parasites of great economic importance, as some species can affect the fish fillet quality by producing macroscopic cysts or generating post mortem myoliquefaction, commonly referred to as ‘soft flesh’. *Kudoa mirabilis* is a ‘soft flesh’-inducing species originally described based on morphology in the musculature of *Trichiurus lepturus* from the Indian Ocean. An integrative morphological and genetic characterization of *K. mirabilis* from the type host caught off the coast of Tanzania is here provided. The spores were stellate with four unequal polar capsules, showing similarities to *Kudoa thyrsites*. For comparative and validation purpose, *K. mirabilis* was compared morphologically and genetically with *K. thyrsites* reference isolates, including new obtained samples from the type host *Thyrsites atun* caught in the SE Atlantic Ocean. Morphological analyses of spores revealed key diagnostic characters clearly distinguishing the two *Kudoa* species. Phylogenetic analyses based on SSU and LSU rRNA genes demonstrated that *K. mirabilis* is a distinct and valid species, representing a sister group to a *K. thyrsites* subclade that comprises several isolates from Japan and one single isolate from South Africa. This finding raises questions about the true diversity likely hidden in the *K. thyrsites* complex.

## 1. Introduction

Myxosporeans (Cnidaria, Myxozoa) are microscopic endoparasites commonly found in marine and freshwater fish [1,2,3]. The genus *Kudoa* Meglitsch, 1947 currently consists of around 100 nominal species described from a wide range of fish host species and geographical areas [1,4,5,6]. The species are histozoic, usually infecting the skeletal musculature of fish, while others occur in the brain, heart, gills, kidney, ovary, or intestines [1,4,5].

Although *Kudoa* spp. are generally not considered pathogenic for the host, several species are of concern to the fishery and aquaculture industries as they may produce unsightly cysts in the fish host’s musculature, or are associated with post mortem myoliquefaction of the fish muscle [1,2,7,8,9,10,11]. The muscle degradation, commonly known as ‘soft flesh’, may irreversibly reduce the quality of the fish fillet and the marketability of the fish product, resulting in economic losses to the seafood industry, as well as loss of consumer confidence [1,4,12] Many *Kudoa* species have been associated with ‘soft flesh’ in commercially valuable wild and cultured marine fish. One of the most conspicuous ‘soft flesh’-inducing species is *Kudoa thyrsites* (Gilchrist, 1924), which infects many marine fish species worldwide including Atlantic mackerel (*Scomber scombrus*) and mahi-mahi (*Coryphaena hippurus*), as well as Atlantic salmon (*Salmo salar*) and olive flounder (*Paralichthys olivaceus*) in mariculture [5,12]. Other ‘soft flesh’-inducing *Kudoa* species found to infect from economically important fishes include *Kudoa musculoliquefaciens* (Matsumoto, 1954) in swordfish (*Xiphias gladius*), *Kudoa paniformis* Kabata and Whitaker, 1981, in Pacific hake (*Merluccius productus*) and *Kudoa rosenbuschi* (Gelormini, 1943) infecting Argentine hake (*Merluccius hubbsi*) [5,12].

The typical life cycle of myxosporeans, involves annelids (e.g., oligochaetes and polychaetes) as invertebrate hosts and fish as vertebrate hosts [13,14]. In the annelid, sexual processes culminate in the production of actinospores. These are infective to the vertebrate host, where another cycle of sporogony results in the production of myxospores. There is evidence for the presence of waterborne infectious stages (actinospores) in *K. thyrsites* [15], but no full life cycle is known in the genus. 

*Kudoa* spp. are characterized by the myxospore stage, composed of four or more shell valves, each associated with a polar capsule [4]. In the past, *Kudoa* spp. identification was based on myxospores morphology, i.e., shape and dimensions of the spore, polar capsules, and polar filaments [16]. More recently, DNA sequence data (mainly rRNA genes) has become increasingly important in the description and species delimitation of new *Kudoa* spp., revealing a large diversity within the genus and demonstrated the existence of cryptic species [2,17,18,19,20].

Prior to the use of DNA, *Kudoa* species with a stellate spore shape and one polar capsule larger than the others, were likely to be assigned to *K. thyrsites*. With the availability of DNA sequence data, it now appears likely that *K. thyrsites* is a complex of cryptic species, supported by large morphological variation and wide host and geographic range [1,2,21]. Phylogenetic analyses based on several molecular markers (rDNA and nDNA) indicated that on a global scale a certain level of genetic sub-structuring exists between the *K. thyrsites* isolates, suggesting the existence of a species complex with four major regional ‘strains’ [2]. However, some morphological variation between regional isolates were not congruent with the genetic data [2]. Furthermore, in the last decade, multiple *Kudoa* spp. defined by a *K. thyrsites*-like myxospore morphology have been genetically characterized and found to be distinct phylogenetic lineages. These include seven species defined by stellate shape and four unequal polar capsules, i.e., *Kudoa minithyrsites* Whipps et al., 2003, *Kudoa lateolabracis* Yokoyama et al., 2004, *Kudoa megacapsula* Yokoyama and Itoh, 2005, *Kudoa whippsi* Burger and Adlard, 2010, *Kudoa gunterae* Burger and Adlard, 2010, *Kudoa cheilodipteri* Heiniger et al., 2013 and *Kudoa parathyrsites* Kasai et al., 2016 [10,18,22,23,24,25]. 

In 1991, *Kudoa mirabilis* Naidenova and Gaevskaya, 1991, was described from the skeletal muscle of largehead hairtail (*Trichiurus lepturus*) in the Indian Ocean, off the coast of Yemen [26]. The spores were stellate in apical view, with one of the four polar capsules much larger than the others that were about equal in size. The polar filament in the largest polar capsule (PC) curved as a single loop, whereas it formed four coils in the small PCs [26]. The parasite produced ovoid or spherical pseudocysts that could form blisters filled with spores, on the body surface and inside the skeletal muscle tissue of the fish. In addition, muscle myoliquefaction was associated with the infection. No DNA sequence data were provided in the description of *K. mirabilis* and to date the phylogenetic position of the species is unknown.

The type host of *K. mirabilis* is *T. lepturus,* a coastal benthopelagic fish species inhabiting tropical and temperate waters around the world [27]. This species represents one of the most important and commercially valuable fish resources worldwide [28,29]. It is heavily targeted in small scale and commercial fisheries worldwide [28], being among the 25 commercial fish species with highest landings [29]. In China, Korea, Japan, and Brazil, as well as some countries in West Africa (Nigeria, Ghana, and Ivory Coast), *T. lepturus* represents one of the most valuable emerging local fisheries, contributing to economic growth and livelihood support [30,31,32]. Recent surveys on fish resources in the coastal waters of Tanzania revealed that *T. lepturus* occurs abundantly in this area [33]. The potential economic yield from this emerging fishery resource raises the need of deepening the knowledge of *T. lepturus* parasitofauna, with special emphasis on quality reducing and zoonotic species.

The aim of this study was to characterize *K. mirabilis* from its type host *T. lepturus*, by integrative analysis of morphological and genetic characters. Additionally, we aimed to elucidate the phylogenetic relationships between *K. mirabilis* and the morphologically related *K. thyrsites.*

## 2. Materials and Methods

### 2.1. Samples Collection

A total of 69 largehead hairtail (*T. lepturus*) were sampled during April 2019 in the South-West Indian Ocean by the research vessel “Dr. Fridtjof Nansen” (Institute of Marine Research cruise nr. 2018404), operating within the EAF-Nansen Project. Fish were caught using bottom trawls at 100–600 m depth at two different sampling localities off the coast of Tanzania (9°54′ S 39°52′ E, 7°16′ S 39°41′ E). Prior to examination, total length (TL; cm) and total weight (TW; g) were recorded. The fish were then cool-stored on board and examined for post mortem myoliquefaction at 12, 24, 36, and 48 h after catch. The musculature of all *T. lepturus* was examined by manual muscle texture testing and visual inspection of the muscle appearance [34], i.e., whether the basic segmental myomere structure was intact or not. For a preliminary microscopic analysis on board, two subsamples of muscle tissue were taken from each side fillet of all fish. According to the procedures provided by St-Hilaire et al. [35], the samples were placed on glass slides, moistened with saline water, and then minced with a scalpel blade. The squash preparations were examined in a brightfield microscope (400× magnification) for myxospores. When *Kudoa*-like spores were detected, 3× *g* centrifuge tubes (50 mL) were filled with “soft” muscle tissue from the fish. Samples were stored at −20 °C and shipped frozen to the Institute of Marine Research of Bergen (Norway), for further morphological and molecular analyses.

The present *K. mirabilis* sample (isolate referred hereafter as TNZ) was compared morphologically and molecularly with three *K. thyrsites* isolates obtained from different hosts and geographical areas. These included new sample material of *K. thyrsites* (isolate referred hereafter as NAM) recovered from three myoliquefacted specimens of South African Snoek (*Thyrsites atun*) (representing the type host), caught off North-Western Namibia at 18°14,64′ S 11° 45,012′ E. The South African Snoeks belonged to a batch of 5 specimens caught in October 2017 (Cruise nr. 2017408, “Dr. Fridtjof Nansen”, Institute of Marine Research), and were examined following the same procedures as above. As additional reference, two *K. thyrsites* isolates previously morphologically and molecularly (SSU rDNA) characterized from the Mediterranean Sea (ex *Lepidopus caudatus*) (isolate referred hereafter as MED) and the Norwegian Sea (ex *S. scombrus*) (isolate referred hereafter as NWS) [36], were included, as well. The latter isolates originated from a related trichiurid host of *T. lepturus,* the silver scabbardfish (*L. caudatus*), and the type host of *K. histolytica* (syn. *K. thyrsites*), the Atlantic mackerel (*S. scombrus*).

### 2.2. Morphological Analysis of Spores

Spore measurements and morphological analysis were performed using Nikon Digital Sight DS-L1 measuring tool software on multiple digital images obtained with an Olympus BX51 microscope, at 1000× magnification. Morphological characterization of *Kudoa* spores was based on material collected from multiple infected hosts. In total, 55 spores (30 in apical view, 25 in lateral view) from three infected *T. lepturus*, and 39 spores (30 in apical view, 9 in lateral view) from three *T. atun* were measured in fresh smears, following the recommendations of Lom and Arthur [16] and Levsen et al. [34]. In addition, various allometric characters were recorded as additional specific morphological descriptors [36]. Mean, standard deviation and range of values were calculated for each spore character. All measurements were expressed in μm.

### 2.3. Statistical Analyses

Morphometric and allometric measures of *Kudoa* spp. spores obtained from *T. lepturus* (TNZ) and *T. atun* (NAM) were statistically compared with similarly obtained data from *K. thyrsites* spores. The latter *K. thyrsites* spore measurements belonged to two reference isolates (MED, NWS) previously characterized morphologically and genetically, for which raw data were available [36].

Spore dimensions in apical view were compared using Student’s T-test (paired samples) or one-way ANOVA’s (>2 samples) in Statistica^®^ 13.3.0 (TIBCO Software Inc., CA, USA). Post-hoc Turkey’s HSD tests were used following significant ANOVA’s. Heteroscedastic data (Levene’s tests) were log10 transformed upon analysis. Principal component analysis (PCA) was performed on morphometric and allometric data obtained from spores in apical view, to compare *Kudoa* spp. isolates. Analyses were performed in R 3.6.1 (R Foundation for Statistical Computing, Vienna, Austria) [37] using the rda function in the ‘vegan’ 2.5.2. package [38]. In brief, a co-variance matrix was constructed to calculate an eigenvalue matrix, whose values were used to obtain the positions of the individual samples in the PCA ordination plot. Plots with 95% confidence interval of the standard deviations of the points were generated. Few spores were measurable in lateral view, and therefore spore length (L) was not used in statistical analyses.

### 2.4. Molecular Analysis

DNA was extracted from 40 mg of muscle tissue from each infected *T. lepturus* and *T. atun* by using DNeasy^®^ Blood and Tissue Kit (Qiagen, Hilden, Germany) and following the manufacturer’s instructions. DNA concentration was determined using a NanoDrop^®^ ND-1000 spectrophotometer 3.8 (Nanodrop, DE, USA). Partial small subunit rRNA (SSU) gene was amplified by PCR using a new primer pair, Ksp18SF (5′-GGA TAA CTG TGG TAA ATC TAG AGC-3′) and Ksp18SR (5′-GAG CAA TTA TTA CAA GGC TCA RTC-3′). For the large subunit rRNA (LSU) gene amplification, a new reverse primer Ksp28SR (5′-CAG CTC CAT ACA AGT TTA CAC-3′) was used in combination with Kt28S1F (5′-CAA GAC TAC CTG CTG AAC-3′) [39]. The new primers were designed based on SSU and LSU rDNA sequences of *Kudoa* spp. available in GenBank and verified by means of the on-line software Primer 3 (http://bioinfo.ut.ee/primer3-0.4.0/). PCR reactions were performed in 25 mL volume containing 100 ng template DNA, 0.5 μL of each primer (50 mM) (Thermo Fisher Scientific, MA, USA), 1 μL of MgSO4 (50 mM) (Invitrogen, CA, USA), 2.5 μL of 10× high-fidelity buffer (Invitrogen), 2 μL of dNTPs (10 mM) (Invitrogen), 0.2 μL Platinum *Taq* DNA polymerase (Invitrogen), and MilliQ water. The following PCR conditions were used to amplify both SSU and LSU rRNA genes: 95 °C for 4 min (initial denaturation), then 35 cycles of 95 °C for 30 s (denaturation), 64 °C for 45 s (annealing), and 72 °C for 1.30 min (extension), followed by a final elongation at 72 °C for 7 min. Purification and sequencing of PCR products were carried out by Eurofins (Cologne, Germany), using the same primers as the ones used for the amplification. For comparison, longer sequences were generated from *K. thyrsites* isolates previously identified from the Norwegian Sea (NWS) and the Mediterranean Sea (MED) [36]. These were also amplified and sequenced using the primers and procedure above.

### 2.5. Phylogenetic Analyses and Genetic Divergence Estimation

Sequences were assembled using ChromasPro 2.1.5 software (Technelysium Pty Ltd., Tewantin, Australia) and analysed in GenBank database (BLAST, www.ncbi.nlm.nih.gov/BLAST). The present sequences were aligned with SSU or LSU rDNA *Kudoa* spp. sequences downloaded from GenBank using ClustalX 2.0 software [40]. High similarity scores in the Basic Local Alignment Search Tool (BLAST) as well as spore morphology, tissue tropism, infected host family, and geographic distribution, were used as the criteria to select the sequences. The default setting parameters of ClustalX 2.0 was used, and the alignments were manually edited and trimmed with BioEdit 7.0.5.3 [41]. The SSU and LSU alignments comprised 1298 characters and 39 taxa, and 816 characters and 48 taxa, respectively. *Kudoa islandica* Kristmundsson and Freeman, 2014, was used as outgroup taxon in both rDNA sequences datasets.

Phylogenetic analyses based on the SSU and LSU rDNA sequences were carried out using both Bayesian inference (BI) and maximum likelihood (ML) methods. The optimum evolutionary model for each rDNA dataset was estimated using the Bayesian information criterion (BIC) and Akaike information criterion (AIC) as implemented in jModeltest 2.1.7 [42].

General time-reversible model with a proportion of invariable sites and a gamma-distributed rate variation across sites (GTR + I + G) was selected as the best-fit evolutionary model for both rDNA datasets, in ML and BI analyses. ML analyses were performed in MEGA X v10.1.6 [43], and nodal support was inferred based on 1000 bootstrap replicates. BI analyses were conducted in MrBayes 3.1.2 [44]. Posterior probability distributions were generated using the Markov Chain Monte Carlo (MCMC) method with four chains, being run simultaneously for 1,000,000 generations. Burn in was set at 0.25 in fraction and trees were sampled every 100 generations, making a total of 7500 trees used to compile the majority rule consensus trees. The above settings were used for all SSU and LSU rDNA phylogenetic trees analysis. Consensus trees were created and visualized using FigTree v1.4.2 (http://tree.bio.ed.ac.uk/software/figtree/).

Divergence estimation matrices were calculated in MEGAX using the Kimura 2-parameter model. The analysis was carried out on SSU and LSU rDNA datasets detailed above. Estimates of average divergence over all sequence pairs between groups of taxa were calculated as number of base substitutions per nucleotide site. The rate of variation among sites was modelled with a gamma distribution (shape parameter = 0.5). All ambiguous positions were removed for each sequence pair (pairwise deletion).

## 3. Results

### 3.1. Incidence of Post Mortem Myoliquefaction

The 69 specimens of largehead hairtail (*T. lepturus*) examined had a mean length (±SD) of 90 ± 14 cm and a mean weight (±SD) of 409 ± 179 g. Of the 69 fish, 3 (4.3%) showed signs of myoliquefaction. Specifically, in the earliest stage of examination (i.e., 12 and 24 h after catch), muscle tissues of all the fish were clearly intact. First signs of abnormally soft and liquefied texture were observed at 36 h of cool storage, in three out of 69 *T. lepturus*. No additional cases were recorded at 36 and 48 h. The lengths and weights of the 3 affected fish were 93, 116, and 100 cm and 354, 977, and 564 g, respectively. During microscopical examination of squash preparations from all 69 fish, only these 3 showed *Kudoa* spores.

The South African snoek (*T. atun*) samples consisted of five fish (mean ± SD total length and weight: 95 ± 13 cm, 300 ± 100 g), of which three (60%) showed clear signs of muscle liquefaction approximately 24 h post catch. Total length and total weight of the affected fish was 96, 76, and 104 cm and 3226, 1575 and 3910 g, respectively. Subsequent microscopy of fresh muscle tissue smears from each affected *T. atun* revealed the presence of numerous *Kudoa* spores.

### 3.2. Descriptions of Spores

The spore measurements and allometric data (mean ±SD, range) of *K. mirabilis* infecting *T. lepturus* from the South-West Indian Ocean and *K. thyrsites* infecting *T. atun* off the coast of Namibia, *L. caudatus* from the Mediterranean Sea, and *S. scombrus* from Norwegian Sea, are given in Table 1.

#### 3.2.1. Spore Morphology of *K. mirabilis* (TNZ)

**Figure 1:**
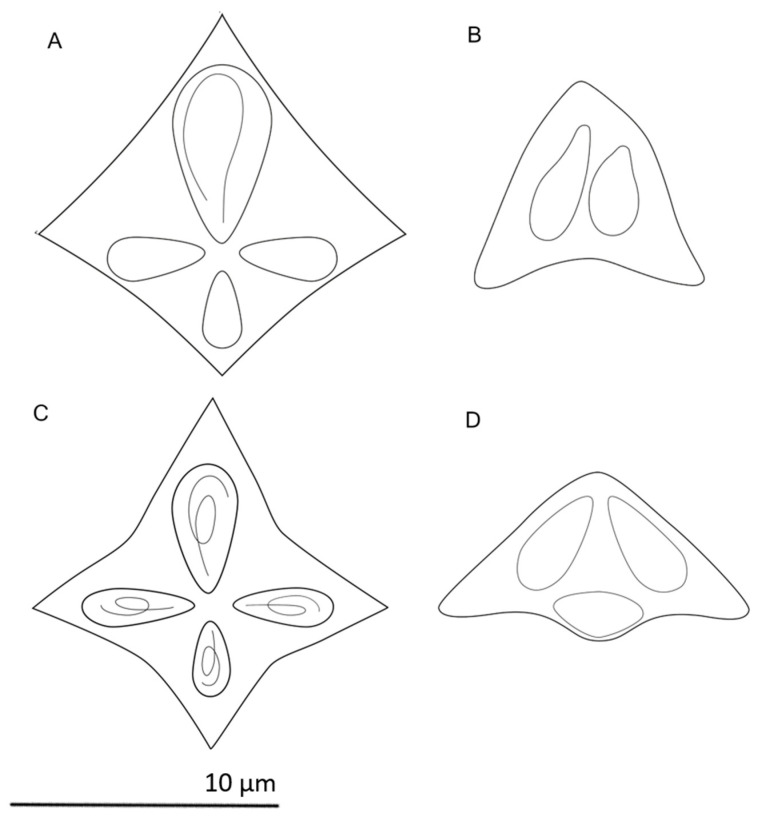
Line drawing of two *Kudoa* spp. in apical and lateral view. (**A**,**B**) *K. mirabilis* in *Trichiurus lepturus* from Tanzanian waters, (**C**,**D**) *K. thyrsites* in *Thyrsites atun* off the coast of Namibia.

In apical view, mature spores were stellate in shape, with four polar capsules (PCs) within thin-walled valves (Figure 1A). The largest PC was much bigger than the other three PCs, occupying much of the volume of the spores. The other three PCs appeared similar to each other in size. However, the PC opposite the large PC was significantly shorter than the other two (T = 5.9, *p* < 0.001). These PC’s are hereafter referred to as “small” and “intermediate”. The polar filament within the large PC curved into a large loop (Figure 1A), while in the smaller PC’s the filament structure could not be clearly seen. In lateral view, the spores were subconical in shape, with pointed posterior apices of the valves (Figure 1B). The spore measurements and allometric data are given in Table 1. The overall spore morphology was similar to the original description of *K. mirabilis* from *T. lepturus* in the Indian Ocean [26].

#### 3.2.2. Spore Morphology of *K. thyrsites* (NAM)

In apical view, mature spores were stellate in shape, with four unequal polar capsules (PCs), each within thin-walled valves (Figure 1C). The spores were characterized by one large, one small, and two intermediate PCs, directed apically to one another. Polar filaments with a single full coil were observed within each PC (Figure 1C). In lateral view, spores were subconical in shape, with posterior apices of valves forming lanceolate processes (Figure 1D). The spore morphometric and allometric data are given in Table 1. The overall spore morphology was similar to the original description of *K. thyrsites* from *T. atun* in South Africa [45].

### 3.3. Morphometric Comparison of the Kudoa spp. spores

**Figure 2:**
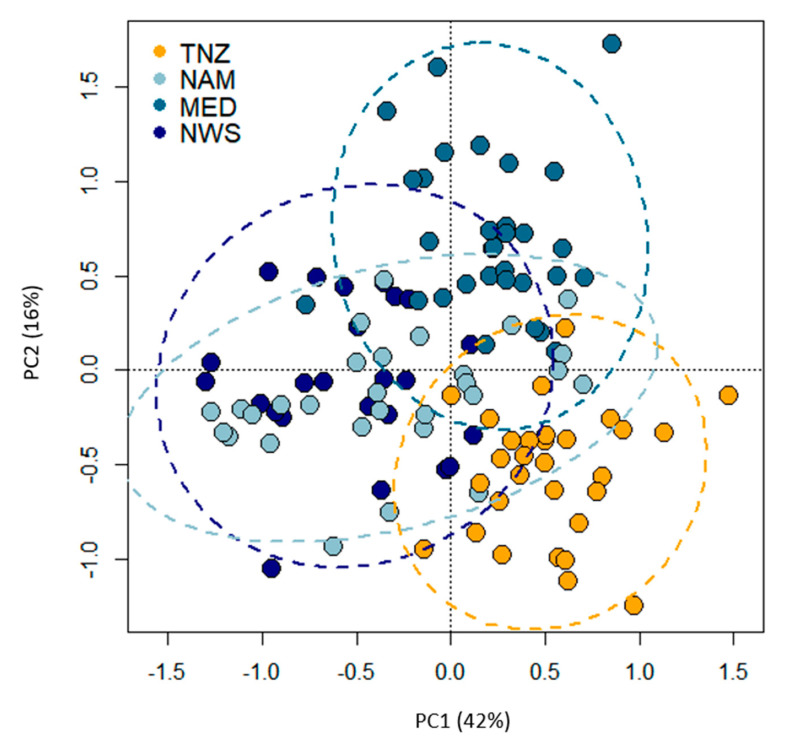
Principal component analysis (PCA) performed on apical view spore measurements of *Kudoa mirabilis* in *Trichiurus lepturus* from Tanzanian waters (TNZ = yellow dots), *Kudoa thyrsites* in *Thyrsites atun* off the coast of Namibia (NAM = light blue dots), *K. thyrsites* in *Lepidopus caudatus* from the Mediterranean Sea (MED = turquoise dots), and *K. thyrsites* in *Scomber scombrus* from Norwegian Sea (NWS = blue dots). Plots with 95% confidence interval of the standard deviations of the points were generated. PC1 and PC2 explains approximately 42% and 16%, respectively, of the observed variability.

A PCA based on the morphometric and allometric data from apical view of the four *Kudoa* isolates showed high overlap between them (Figure 2). However, there was separation of *K. mirabilis* along PCA1/2 axes from the three *K. thyrsites* isolates.

**Figure 3:**
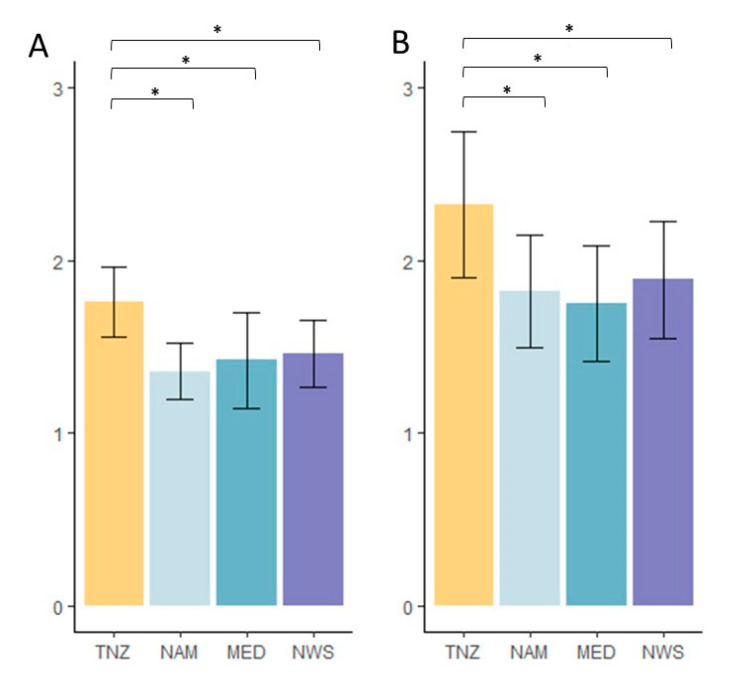
Bar-plot representation of two spores allometric measurements distribution, (**A**) LPCL: IPCL and (**B**) LPCL: SPCL, of *Kudoa* spp. examined. *K. mirabilis* in *Trichiurus lepturus* from Tanzanian waters (TNZ = yellow), *K. thyrsites* in *Thyrsites atun* off the coast of Namibia (NAM = blue), *K. thyrsites* in *Lepidopus caudatus* from the Mediterranean Sea (MED = turquoise), and *K. thyrsites* in *Scomber scombrus* from Norwegian Sea (NWS = blue). Asterisks indicate significant differences, according to one-way analyses of variance (ANOVA). Significance was fixed at *p* < 0.05.

Statistical analyses (ANOVA) of morphometric and allometric characters showed that *K. mirabilis* spores did not differ significantly from *K. thyrsites* (NAM, NWS, and MED) in spore size (W1, W2, T1, and T2), spore size-ratios (W1:W2, T1:T2), or polar capsule size (LPCL, IPCL, and SPCL). However, the ratios between the length of large PC and the others (LPCL: IPCL, LPCL: SPCL) differed significantly between *K. mirabilis* and *K. thyrsites* (Figure 3A,B).

### 3.4. Molecular Identification

Partial SSU and LSU rDNA (1287 bp and 1454 bp, respectively) sequences were obtained from *K. mirabilis* (TNZ) spores sampled from liquefied muscle of the three individual *T. lepturus* from Tanzanian waters. The SSU sequences from the three individual fish were identical, while one of the LSU sequences differed at a single site (K: G or T). The SSU sequences showed highest identity (99.3%, 1278/1287 bp) with *K. thyrsites* isolated from black scraper (*Thamnaconus modestus*) and olive flounder (*Paralichthys olivaceus*) in Japan (GenBank accession nos. LC128644, LC128645, and AY382607).

The LSU rDNA *K. mirabilis* sequences showed highest identities with *K. thyrsites* isolated from South African hake (*Merluccius capensis*) in South Africa (95.4%, 1389/1456 bp), *T. modestus* (93.7%, 1386/1479 bpand 93.6%, 1394/1489 bp), and *P. olivaceus* in Japan (93.7%, 1395/1489 bp) (GenBank accession nos. AY941819, LC128644, LC128645, JQ302300). The partial SSU and LSU sequences of *K. mirabilis* are deposited in GenBank under the accession numbers MT919693 and MT919732, MT919733, respectively.

The partial SSU and LSU rDNA sequences (1289 bp and 1446 bp, respectively) generated from *K. thyrsites* (NAM) spores sampled from liquefied muscle of the three individual *T. atun* from Namibia were all identical. The SSU sequences matched 100% (1287/1287 bp) with *K. thyrsites* isolated from the *T. atun* and *M. capensis,* in South Africa (GenBank accession nos. AY078430 and AY941819). The LSU sequences showed highest identity (99.9%, 1444/1446 bp) to *K. thyrsites* from *M. capensis* fished in South Africa (GenBank accession nos. AY941819). The partial SSU and LSU sequences of *Kudoa thyrsites* from *T. atun* were deposited in GenBank under the accession number MT912846 and MT919735, respectively.

Additional partial SSU and LSU rDNA sequences (1289 bp and 1446 bp, respectively) were generated from two *K. thyrsites* isolates previously identified from *L. caudatus* and *S. scombrus*, caught in the Mediterranean Sea and Norwegian Sea, respectively [36]. *K. thyrsites* isolate obtained from eight *S. scombrus* (NWS) had identical SSU rDNA sequences, while LSU rDNA sequences showed one substitution. No nucleotide variations were observed in SSU and LSU sequences of *K. thyrsites* isolate from the two *L. caudatus* (MED). These new partial SSU and LSU sequences of *K. thyrsites* were deposited in GenBank under the accession number MT912847, MT913636 and MT919734, MT919736, and MT919737, respectively.

### 3.5. Phylogenetic Position and Genetic Divergence

**Figure 4:**
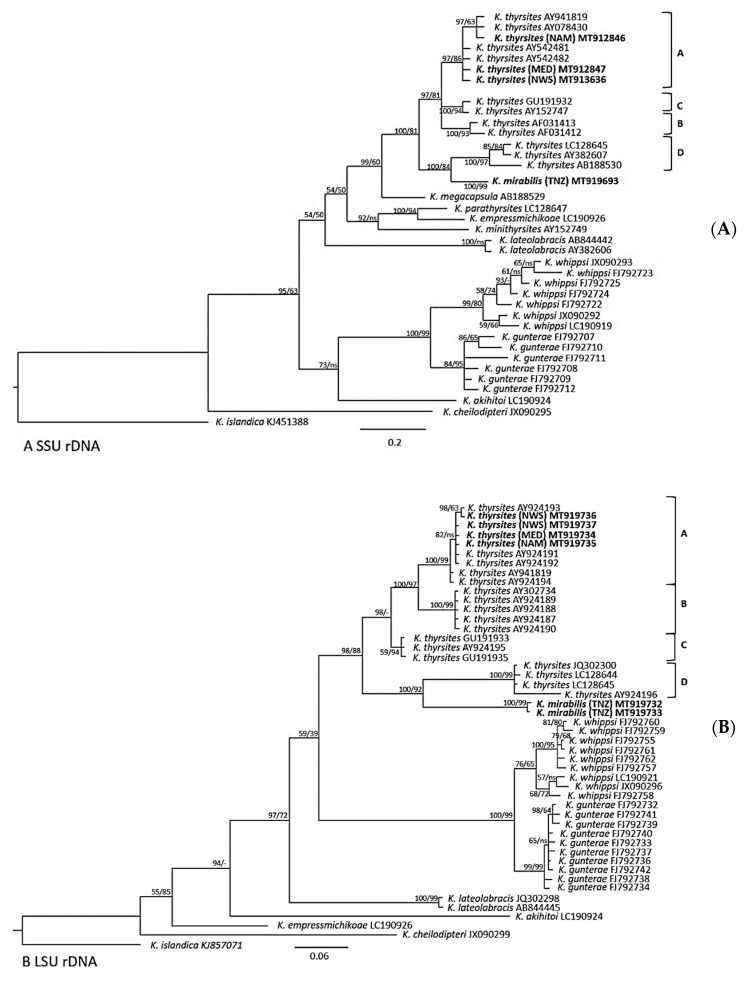
Bayesian inference (BI) phylogenetic tree based on sequences of (**A**) SSU rDNA dataset and (**B**) LSU rDNA dataset. Species from this study are shown in bold. Locality of each new isolates obtained from this study are listed to the right with the following abbreviations: TNZ, (*Kudoa mirabilis* ex *T. lepturus* from Tanzanian waters); NAM, (*Kudoa thyrsites* ex *T. atun* from Namibian waters); MED, (*K. thyrsites* ex *L. caudatus* from Mediterranean Sea); and NWS, (*K. thyrsites* ex *Scomber scombrus* from Norwegian Sea). GenBank accession number follows each taxon. *Kudoa islandica* was set as outgroup. Nodal supports are indicated for BI (posterior probabilities) and ML (bootstrap, *n* = 1000); Nodal values with < 50 support are indicated by dashes (-); (ns) denotes a different branching for the ML tree. There are two *K. thyrsites* clade identified robustly supported: the major clade, including isolates from Atlantic Ocean, Pacific Ocean, Australia (labelled subclades A–C), and a small subclade, including isolates from Japan and one single isolate from South Africa (in LSU tree) (labelled subclade D). The latter clade formed as a sister lineage to *K. mirabilis*.

Phylogenetic analyses included 39 SSU and 48 LSU rDNA *Kudoa* spp. sequences available from GenBank, as well as the 12 sequences (6 for each rRNA gene) generated in this study. Phylogenetic trees of similar topology were obtained for both SSU and LSU rDNA datasets using Bayesian and Maximum likelihood methods (Figure 4A,B). All phylogenetic analyses placed *K. mirabilis* (TNZ) within a clade comprising muscle infecting *Kudoa* species with stellate spore shape and four unequal polar capsules. This clade—named “*thyrsites*-like morphotype clade” by Heininger et al. [24] (p. 201)—includes *K. thyrsites, K. mirabilis, K. megacapsula, K. parathyrsites, K. empressmichikoae, K. minithyrsites, K. lateolabracis, K. whippsi, K. gunterae,* and *K. akihitoi*.

In both SSU and LSU analyses, *K. mirabilis* (TNZ) represented a distinct lineage in sister relationship with a *K. thyrsites* subclade which includes sequences originating from Japan and South Africa (subclade D, Figure 4A,B). *K. mirabilis* (TNZ) and *K. thyrsites* subclade D represented a sister group to a major *K. thyrsites* clade comprised of subclades A, B, and C from several different geographical areas (subclade A, Atlantic Ocean; subclade B, Pacific Ocean; subclade C, Australia, Figure 4A,B). *K. thyrsites* (NAM) from the type host *T. atun* clustered together with other *K. thyrsites* isolates from the Atlantic Ocean (subclade A, Figure 4A,B), including the new sequences (MED, NWS) obtained here from *L. caudatus* and *S. scombrus.*

Genetic divergences in SSU and LSU rDNA between *K. mirabilis* (TNZ) and phylogenetically closely related taxa are shown in Table 2 According to the existence of four geographical subclades of *K. thyrsites*, its relative sequences were divided into four corresponding groups: Atlantic Ocean (subclade A), Pacific Ocean (subclade B), Australia (subclade C), all included in the major clade, and Japanese (plus one sequence from South Africa, exceptionally present here) clustering as a secondary subclade (D) (Table 2, Figure 4A,B). Each group contained all the corresponding sequences reported in the phylogenetic trees (Figure 4A,B). In pairwise comparisons, the average genetic divergence of SSU and LSU rDNA between *K. mirabilis* (TNZ) and *K. thyrsites* Japanese/South African subclade (subclade D), were 0.0077 and 0.0981, respectively. Similarly, the genetic divergence of SSU and LSU rDNA between *K. mirabilis* (TNZ) and the other three subclades of *K. thyrsites* (subclades A, B, and C) ranged between values 0.00870–0.0099, and 0.08080–0.0867, for each locus, respectively. Consistently, *K. thyrsites* subclades in the major clade (subclades A, B, and C) showed a similar level of genetic divergence for SSU and LSU rDNA sequence from the *K. thyrsites* Japanese/South African subclade (subclade D) with approximately 0.009 and 0.08 values. Genetic divergence of SSU and LSU rDNA among the three *K. thyrsites* subclades within the major clade (subclades A, B, and C) where lower than those above mentioned (see Table 2).

The average genetic divergence of SSU and LSU rDNA between *K. whippsi* and *K. gunterae* were 0.0092 and 0.0389, respectively, and were 0.0063 for SSU between *K. parathyrsites* and *K. empressmichikoae* (Table 2). For the latter two species, no comparable bp-size sequences were available to estimate genetic divergence of LSU rDNA. All other analysed taxa presented genetic divergence greater than or equal to 0.009.

## 4. Discussion

Members of the genus *Kudoa* are fish parasites of great economic importance, as some species can affect the fish fillet quality by producing macroscopic cysts or generating post mortem myoliquefaction [1,5,9]. Despite this, little is known about their biology and ecology. Infections tend to be noted in heavily infected hosts (likely abnormal cases), and life cycle is unknown.

Traditionally, the identification of *Kudoa* spp. relied on morphological characters of myxospores [46]. However, the simple morphology of the spore-stage provides few suitable characters. Moreover, the intraspecific variability in spore size and morphology, and the interspecific convergence of characters pose challenges in discriminating *Kudoa* species based only on morphology [47]. In the last two decades, more robust species characterizations became possible through integrative analysis of multiple characters including molecular data, spore morphology, tissue tropism, host specificity, geographical distribution, and clinical signs [18,24,39,48,49,50]. However, recent studies demonstrated that molecular and morphological data are not always congruent, and that some characters are more informative than others [47]. For instance, tissue tropism appears to be an important biological character which reflects some major phylogenetic relationships in the Kudoidae [49], while host range and morphological characters do not always correspond to the molecular phylogeny [51].

In this study, an integrated morphological and genetic characterization of *K. mirabilis* is provided, together with new data on *K. thyrsites.* The results obtained show that *K. mirabilis* is a valid species, morphologically characterized by a distinctive relationship between the size of the polar capsules. *K. mirabilis* proved genetically distinct but phylogenetically related to some myxosporeans identified with *K. thyrsites* (subclade D).

Microscopic examination conducted on myoliquefacted muscle tissue of three largehead hairtail (*T. lepturus*) from South-West Indian Ocean revealed the presence of *Kudoa* spores. Overall morphology and morphometry of the spores showed closest resemblance to *K. mirabilis* originally described from the musculature of *T. lepturus* caught off Yemen (Indian Ocean) [26]. The most conspicuous character was the stellate shape of the spores, the prominence of the largest PC and the loop-like appearance of the polar filament in this capsule. The same host, general geographic area, and ability to cause post mortem myoliquefaction strongly support conspecificity. However, clusters of pseudocysts forming blisters in the flesh of the originally examined fish, were not recorded in this study, possibly because the present infections were lighter. The typical polar filament arrangement in the large PC, folded in a wide loop, was confirmed in this study. However, the four coiled filaments in the smaller polar capsules could not be confirmed in the samples here examined (not observed). Despite the close similarities in the overall morphology and morphometry, it appears that the PC lengths recorded in this investigation were slightly smaller than those of Naidenova and Gaevskaya [26], while spore width and thickness observed here were larger than in the original description. This variation in spore size is probably due to the heterogeneity of measuring techniques adopted by different authors [36]. For instance, PCs were likely measured in lateral view in the original description while here were taken in apical view, representing the PC span. Moreover, the long and short axes (W1, T1; W1, W2) were not distinguished in the original description.

Naidenova and Gaevskaya [26] did not discern small and intermediate PCs, but their images suggest that one PC is slightly smaller. Indeed, the size difference between small and intermediate PCs found in the present study was also quite small. The allometric ratios between the length of large PC and the others (LPCL: IPCL, LPCL: SPCL) in the original description of *K. mirabilis* were remarkably similar to those calculated in the present study. Indeed, in the original description the ratio between the large and the small capsules ranged 1.7–2, showing similar values to those observed here (LPCL: IPCL, 1.5–2.2; LPCL: SPCL, 1.7–3.6). The overall spore morphology and morphometry of *K. mirabilis* (TNZ) were compared with three geographical isolates (NAM, MED, and NWS) of the sister species *K. thyrsites* (see Table 1). These represent isolates from the type host of *K. thyrsites* (NAM), from a related trichiurid host of *T. lepturus*, the silver scabbardfish (*L. caudatus*) (MED), and from the type host of *K. histolytica* (syn. *K. thyrsites*), the Atlantic mackerel (*S. scombrus*) (NWS). Despite similarities in overall shape and appearance, the proportions between the capsule dimensions, as well as the polar filament arrangement, differed between the *K. mirabilis* and *K. thyrsites*. Principal component analyses (PCA) indicated some differentiation between spores corresponding to the two *Kudoa* species (Figure 3). Significant differences were recorded between *K. mirabilis* and *K. thyrsites* in LPCL: IPCL and LPCL: SPCL ratios; thus, clearly differentiating the two species.

In contrast, width, thickness, and PC lengths—previously found of diagnostic value for distinguishing between numerous similar morphological species (e.g., *K. whippsi*, *K. gunterae*, *K. parathyrsites*, *K. minithyrsites*, *K. lateolabracis,* and *K. cheilodipteri*) [10,18,22,24,25]—did not differ. These findings indicate the importance of the allometric spore measurements as key diagnostic characters in *Kudoa* spp. species identification [36].

In the phylogenetic analyses based on SSU and LSU rDNA sequences, *K. mirabilis* clearly represented a distinct lineage and a sister group to a well-supported *K. thyrsites* subclade (subclade D, Figure 4A,B). This subclade comprised of several isolates from Japan, and one from South Africa. *K. mirabilis* (TNZ) and subclade D represented a robustly supported sister group to the major *K. thyrsites* clade (Figure 4A,B). The major clade was composed by *K. thyrsites* isolates originating from different localities and grouped in three main distinct subclades, corresponding to three broad geographical areas (i.e., Atlantic Ocean, Pacific Ocean, and Australia, subclades A–C, Figure 4A,B). In addition, the phylogenetic tree topology (MP and BI) inferred from SSU and LSU rRNA genes clearly demonstrated that *K. mirabilis* formed a monophyletic clade with *K. thyrsites*, suggesting the existence of a common ancestor.

An alternative interpretation could be that *K. mirabilis* is a geographical variant of *K. thyrsites*, rather than a distinct species. However, the genetic divergence between *K. mirabilis* and its closest relative in subclade was 0.0077 and 0.0981, respectively. Similar values of genetic divergence were also calculated between *K. mirabilis* (TNZ) and the other three *K. thyrsites* subclades from the major clade (Atlantic Ocean, Pacific Ocean, and Australia), ranging between values 0.0087 and 0.0099, and 0.0808 and 0.0867, for SSU and LSU rDNA, respectively (subclades A–C, Table 2). The genetic divergence of SSU and LSU rDNA between *K. mirabilis* (TNZ) and the four *K. thyrsites* clades were also similar to those observed between other valid species of *Kudoa* such as between *K. whippsi* and *K. gunterae*, or *K. parathyrsites* and *K. empressmichikoae*. Moreover, the genetic divergence between *K. mirabilis* (TNZ) and *K. thyrsites* observed in this study is in accordance with the typical level of interspecific variation of SSU and LSU rDNA in myxosporeans, i.e., >0.02 [19,52,53,54]. Therefore, it can be concluded that the genetic divergence values between *K. mirabilis* (TNZ) and *K. thyrsites* is assessed at interspecific level, providing further evidence that they represent two distinct species. Clearly, genetic divergence alone does not define species, but in concordance with phylogenetic and morphological evidences it may aid species delimitation [18].

The recognition of *K. mirabilis* as a unique and genetically distinct lineage with respect to the sister Japanese/South African subclade of *K. thyrsites* (Group D, Figure 4A,B) raises questions on the specific boundaries in the *K. thyrsites* complex. The subclade D of *K. thyrsites* could have been regarded as a geographical (i.e., Japanese) variant, if not for the single sequence from a South African *Beryx splendens* [23]. Hence, this genotype may occur in sympatry with *K. thyrsites* sensu stricto from *T. atun* (i.e., type host and general geographic area) and some other fish from Southern Africa, rendering conspecificity of these *K. thyrsites* genotypes unlikely. Whipps and Kent [2] conducted a phylogeographic study of *K. thyrsites* using SSU rDNA, internal transcribed spacer 1 (ITS-1), LSU rDNA, and heat shock protein 70 (hsp70) gene sequences. The evidence suggested that *K. thyrsites* may represent a complex of cryptic species, albeit not conclusively [2]. Indeed, they did not find distinctive morphological characters, or other biological and ecological traits, consistent with the genetically recognized strains [2,18,47]. Later, more *K. thyrsites* isolates were identified worldwide, particularly in the Mediterranean Sea and in the waters around Australia and off Japan [11,24,36]. These and the present observations tend to increase the *K. thyrsites* diversity, supporting the view of Whipps and Kent [2]. In particular, the evidence from this study suggest that the members of clade D of *K. thyrsites* actually represent an unrecognized sibling species.

In conclusion, this study has shown that *K. mirabilis* is a valid species and represents a sister species of the morphologically similar *K. thyrsites*; thus, sharing a common evolutionary history. The herein discovered sister relationship between *K. mirabilis* and the Japanese/South African *K. thyrsites* subclade (group D), as well as their relative position and difference in genetic divergence values to the major *K. thyrsites* clade, strongly suggests that *K. thyrsites* is a complex of cryptic species with still unresolved diversity and phylogeny [2]. To disentangle these aspects, additional characters would be valuable, such as mtDNA gene sequences and ultimately life cycle characteristics.

## Figures and Tables

**Table 1:** Spore morphometry of *Kudoa mirabilis* infecting *Trichiurus lepturus* from the South-West Indian Ocean (TNZ) and *K. thyrsites* isolates infecting *Thyrsites atun* off the coast of Namibia (NAM), *Lepidopus caudatus* from the Mediterranean Sea (MED) and *Scomber scombrus* from Norwegian Sea (NWS). Measurements are given in µm as mean, standard deviation, and range in parenthesis. W1, Width1; W2, Width2; T1, Thickness1; T2, Thickness2; L, Length; LPCL, Length of large polar capsule; IPCL, Length of intermediate polar capsule; and SPCL, Length of small polar capsule.

Species	*Kudoa Mirabilis*	*Kudoa Thyrsites*	*Kudoa Thyrsites*	*Kudoa Thyrsites*
Locality	Coast off Tanzania, SW Indian Ocean	Coast off Namibia, SE Atlantic Ocean	Coast off Motril, Alboran Sea	Norwegian Sea
Host	*Trichiurus lepturus*	*Thyrsites atun*	*Lepidopus caudatus*	*Scomber scombrus*
N of myoliquefactive fish	*n* = 3	*n* = 3	*n* = 2	*n* = 8
Total length (TL) and total weight (TW) of myoliquefactive fish	(TL, 93 cm; 116 cm; 100 cm; TW, 354 g; 977 g; 564 g)	(TL, 96 cm; 76 cm; 104 cm; TW, 3226 g; 1575 g; 3910 g)	(TL, 135 cm; 121.0 cm; W, 1778 g; 1500 g)	(mean TL, 37 cm; mean TW, 436.5 g)
Reference and sample abbreviation	Present study (TNZ)	Present study (NAM)	Giulietti et al. (2019) (MED)	Giulietti et al. (2019) (NWS)
Number of examined spores (apical/lateral view)	30/25	30/9	30/30	30/30
Morphometric characteristic and ratios	mean ± SD	mean ± SD	mean ± SD	mean ± SD
	(range)	(range)	(range)	(range)
W1	13.6 ± 0.9	13.7 ± 1.0	15.5 ± 1.1	15.6 ± 1.4
	(12.3–16.0)	(11.1–15.5)	(13.0–17.3)	(13–18.7)
W2	12.6 ± 0.8	13.5 ± 0.9	14.7 ± 1.2	14.5 ± 1.3
	(11.1–14.4)	(11.9–16.0)	(12.2–16.8)	(11.7–17.4)
T1	10.4 ± 0.8	10.3 ± 0.9	12.0 ± 1.2	11.8 ± 1.1
	(8.9–12.1)	(8.7–12.6)	(9.5–14.5)	(9.4–14.0)
T2	8.2 ± 0.7	8.9 ± 1.0	9.9 ± 0.9	9.6 ± 1.1
	(6.9–10.0)	(6.7–10.9)	(8.4–12.3)	(6.8–11.4)
L	7.0 ± 1.1	6.6 ± 0.6	7.7 ± 1.0	7.1 ± 1.2
	(5.2–10.7)	(5.8–7.9)	(6.4–11.2)	(5.3–8.0)
LPCL	5.4 ± 0.4	4.4 ± 0.5	5.1 ± 0.7	5.5 ± 0.7
	(3.9–6.2)	(3.5–5.2)	(4.1–6.7)	(4.3–6.9)
IPCL ^a^	3.1 ± 0.4	3.3 ± 0.4	3.7 ± 0.9	3.9 ± 0.8
	(1.8–4.0)	(2.5–4.1)	(2.3–6.0)	(2.4–5.3)
SPCL	2.4 ± 0.5	2.5 ± 0.4	3.0 ± 0.7	3.0 ± 0.7
	(1.4–3.3)	(1.9–3.6)	(1.5–4.5)	(1.9–4.1)
W1/W2	1.1 ± 0.1	1.0 ± 0.1	1.1 ± 0.1	1.1 ± 0.1
	(1.0–1.2)	(0.9–1.1)	(1.0–1.2)	(0.9–1.2)
T1/T2	1.3 ± 0.1	1.2 ± 0.1	1.2 ± 0.2	1.2 ± 0.1
	(1.0–1.6)	(0.9–1.7)	(0.9–1.5)	(1.1–1.6)
LPCL/IPCL	1.8 ± 0.2	1.4 ± 0.2	1.4 ± 0.3	1.5 ± 0.2
	(1.5–2.2)	(1.1–1.7)	(0.8–2.0)	(1.2–2.0)
LPCL/SPCL	2.3 ± 0.4	1.8 ± 0.3	1.8 ± 0.3	1.9 ± 0.3
	(1.7–3.6)	(1.3–2.5)	(1.1–3.0)	(1.5–2.6)

^a^ Combined measurements (mean) of the two intermediate polar capsules of *Kudoa* spp. spores.

**Table 2:** Genetic divergences over comparable size sequence pairs of the SSU and LSU rDNA between *Kudoa mirabilis* and phylogenetically closely related taxa. Each taxon included all the *Kudoa* corresponding sequences shown in the trees (Figure 4A,B). According to the existence of four geographical strains of *K thyrsites*, its relative sequences were assigned to four corresponding groups: Atlantic Ocean (A), Pacific Ocean (B), Australian waters (C), and Japanese (plus one sequence from South Africa, exceptionally present here) (D). Each group contained all the corresponding sequences reported in the tree.

**SSU rDNA**														
		**1**	**2**	**3**	**4**	**5**	**6**	**7**	**8**	**9**	**10**	**11**	**12**	**13**
1	*K. akihitoi*													
2	*K. cheilodipteri*	0.0409												
3	*K. empressmichikoae*	0.0194	0.0351											
4	*K. gunterae*	0.0203	0.0407	0.0229										
5	*K. lateolabracis*	0.0257	0.0343	0.0212	0.0219									
6	*K. megathyrsites*	0.0212	0.0361	0.0161	0.0195	0.0195								
7	*K. minithyrsites*	0.0272	0.0369	0.0153	0.0246	0.0186	0.0136							
8	*K. mirabilis* (TNZ)	0.0203	0.0379	0.0203	0.0186	0.0221	0.0128	0.0186						
9	*K. parathyrsites*	0.0178	0.0343	0.0063	0.0229	0.0196	0.0144	0.0136	0.0169					
10	*K. thyrsites* (A)	0.0206	0.0390	0.0155	0.0223	0.0207	0.0090	0.0147	0.0090	0.0123				
11	*K. thyrsites* (C)	0.0233	0.0369	0.0149	0.0216	0.0216	0.0083	0.0140	0.0087	0.0132	0.0030			
12	*K. thyrsites* (B)	0.0241	0.0373	0.0157	0.0241	0.0225	0.0115	0.0165	0.0099	0.0140	0.0045	0.0043		
13	*K. thyrsites* (D)	0.0198	0.0401	0.0203	0.0214	0.0241	0.0136	0.0189	0.0077	0.0186	0.0097	0.0098	0.0094	
14	*K. whippsi*	0.0222	0.0407	0.0238	0.0092	0.0258	0.0211	0.0264	0.0196	0.0238	0.0242	0.0234	0.0260	0.0229
**LSU rDNA**														
		**1**	**2**	**3**	**4**	**5**	**6**	**7**	**8**	**9**	**10**			
1	*K. thyrsites* (A)													
2	*K. lateolabracis*	0.0909												
3	*K. thyrsites* (B)	0.0195	0.0977											
4	*K. thyrsites* (C)	0.0337	0.0859	0.0322										
5	*K. thyrsites* (D)	0.0786	0.1376	0.0742	0.0820									
6	*K. gunterae*	0.1177	0.1210	0.1213	0.1026	0.1496								
7	*K. whippsi*	0.1247	0.1319	0.1271	0.1143	0.1492	0.0389							
8	*K. cheilodipteri*	0.1607	0.1775	0.1593	0.1590	0.1818	0.1746	0.1798						
9	*K. akihitoi*	0.1367	0.1858	0.1384	0.1554	0.1768	0.2009	0.2116	0.2352					
10	K. *empressmichikoae*	0.0893	0.1154	0.1013	0.1045	0.1486	0.1613	0.1583	0.1548	0.1518				
11	*K. mirabilis* (TNZ)	0.0867	0.1423	0.0808	0.0809	0.0981	0.1647	0.1595	0.1589	0.1676	0.1336			
